# One-year trial evaluating the durability and antimicrobial efficacy of copper in public transportation systems

**DOI:** 10.1038/s41598-024-56225-9

**Published:** 2024-03-21

**Authors:** Teresa C. Williams, Edouard Asselin, Tony Mazzulli, Tracey Woznow, Hadi Hamzeh, Davood Nahkaie, Dean Waisman, Biljana Stojkova, Richard Dixon, Elizabeth Bryce, Marthe Charles

**Affiliations:** 1 Division of Medical Microbiology and Infection Prevention, Vancouver Coastal Health, Vancouver, BC Canada; 2 Department of Materials Engineering, University of British Columbia, Vancouver, BC Canada; 3 Department of Microbiology, Mount Sinai Hospital, Toronto, Canada; 4 Westech Systems, Inc, Vancouver, Canada; 5 Department of Statistics, University of British Columbia, Vancouver, BC Canada; 6 Community & Healthcare Acquired Infection Reduction (CHAIR), Vancouver, Canada; 7 Division of Medical Microbiology and Infection Prevention, Vancouver General Hospital, 1116 – 855 West 12th Avenue, Vancouver, BC V5Z 1M9 Canada

**Keywords:** Public health, Antimicrobial, Copper, Transit, Bacteria, Microbiology, Environmental sciences, Public health

## Abstract

Surfaces on transit vehicles are frequently touched and could potentially act as reservoirs for micro-organism transmission. Regular cleaning and disinfection to minimize the spread of micro-organisms is operationally challenging due to the need to keep vehicles in circulation. The application of copper (Cu) alloys to high- touch surfaces could help reduce the risk of cross-contamination, however, little is known about the durability and efficacy of engineered copper surfaces after prolonged use. Three Cu products (decal, thermal fabrication, and alloy covers) were assessed over a 12-month period. These Cu products were randomly installed on 110 stanchions on three buses and four train (SkyTrain) cars in Vancouver and three buses, two subway cars, and two streetcars in Toronto with mirrored control surfaces directly opposite. Bacterial counts (Colony forming units, CFU) and ATP bioluminescence (ATPB) were measured every two months after peak morning routes. Durability of the Cu products were assessed monthly through visual inspection and colorimetry assays or by ex-situ microscopy. Cu products on stanchions reduced the mean colony forming units (CFU) of all vehicles by 42.7% in the mean CFU (0.573 (CI 95% 0.453–0.726), p-value < 0.001) compared to control surfaces. The three Cu products exhibited an overall 87.1% reduction in the mean ATPB readings (0.129 (CI 95% 0.059–0.285, p-value < 0.001) compared to controls. Surface Cu concentration for all three products was consistent throughout the 12-month period. Electron microscopy (SEM) and Energy-dispersive X-ray Spectroscopy (EDS) cross-sectional analysis showed no change in thickness or dealloying of Cu products, however SEM top-down analysis revealed substantial carbon accumulation on all surfaces. Cu products installed on transit vehicles maintained antimicrobial efficacy and durability after 12 months of use.

## Introduction

Public transportation systems are an essential mode of transport for many individuals. In 2022, a survey found that one in ten individuals in the United States used public transit on a regular basis and both the Canadian and American transit authorities reported between 1.2 and 5.97 billion annual passenger trips respectively^1,2^. Transit vehicles have multiple surfaces that are frequently touched by users including stop buttons, stanchions, hand grips, and railings. Constant cleaning and disinfection is impossible and the high-touch surfaces have a real risk of becoming reservoirs for bacteria and viruses. The COVID-19 pandemic brought the conversation of environmental reservoir to the forefront and highlighted the importance of implementing innovative antimicrobial mitigation strategies.

For example, a German study investigated antibacterial coatings on transit vehicles over a two-month period and found an absolute risk reduction of 22.6%^3^. The use of copper (Cu), a well-established self-sanitizing substrate has garnered scientific interest particularly with the advent of the COVID-19 pandemic^4^. Historically, Cu has been approved for use in public settings by both the Environmental Protection Agency (EPA) and Health Canada’s Pest Management Regulatory Agency (PMRA), as early as 2008 and 2014 respectively. It has been implemented as an antibacterial surface agent in hospitals and care facilities^5–7^, medical devices^8^, university dorms^9^ and athletic centers^10^ with demonstrated significant reductions in bacterial CFU compared to stainless steel and plastic controls. There is limited data on the long-term efficacy and durability of Cu in high-volume, high-turnover environments, such as public transit systems^11^.

Three different commercially available Cu alloy products ranging from 80 to 91.3% Cu content were installed on high-touch surfaces in buses and train (SkyTrain) cars in Vancouver, as well as subway cars, streetcars and buses in Toronto and monitored over the course of one year. The primary objective of this study was to establish the antimicrobial efficacy and durability of Cu alloy surfaces over 12 months of use in public transit vehicles located in two Canadian cities.

## Methods and materials

### Study design

This study was powered for significance using three commercial Cu products registered with Health Canada PMRA. The products consisted of a Cu alloy formulation (80% Cu, 20% Ni), a Cu-containing decal (91.3% Cu), and a proprietary thermal fabrication Cu coating (approximately 67% Cu, approximately 33% Ni-Zn). The proprietary thermal fabrication process was applied on both stainless steel and a plastic prototype for evaluation on public transit vehicles.

**Figure 1:**
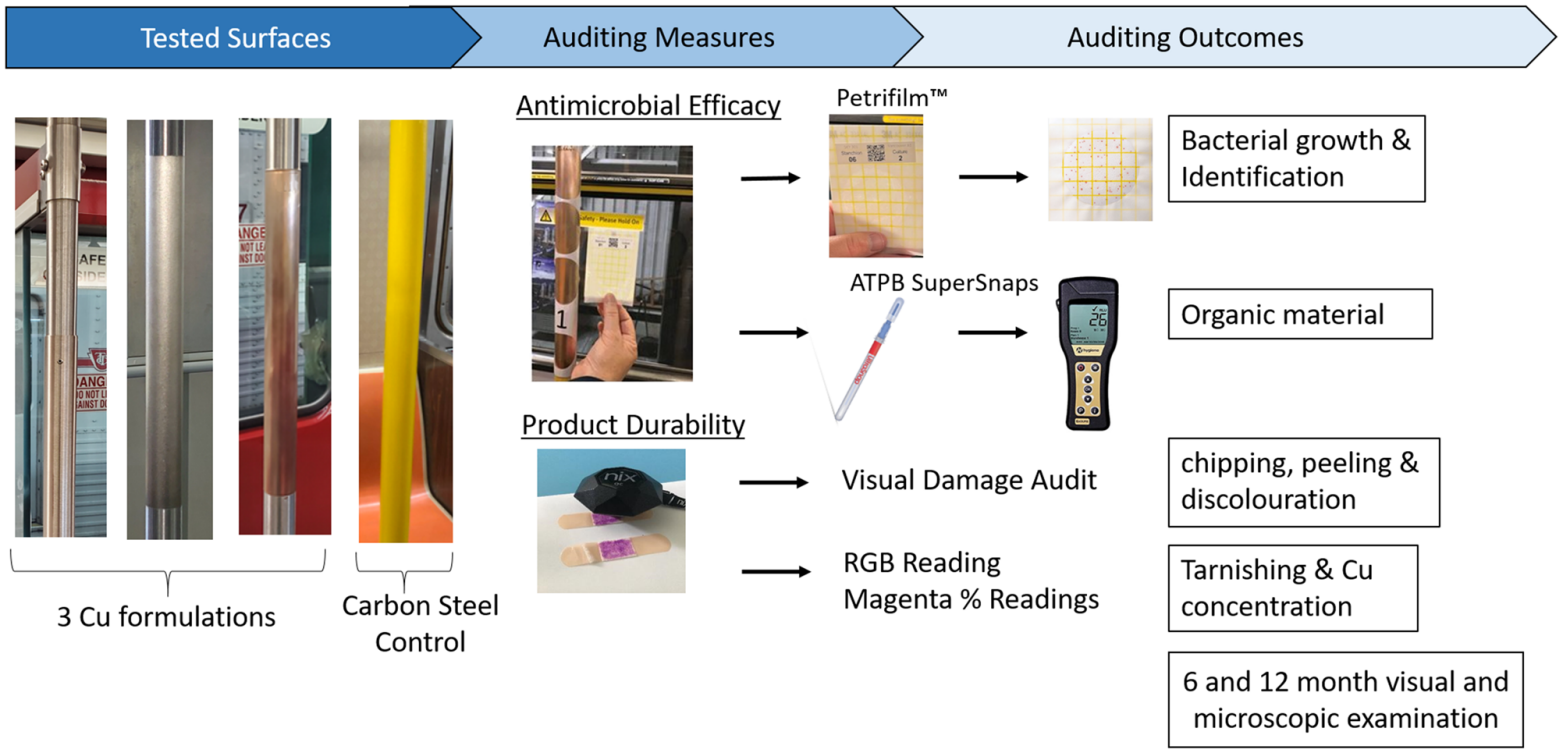
Overview of surfaces, auditing measures, and auditing outcomes for the 12-month in-use trial on public transit vehicles.

The 12-month trial was conducted in collaboration with the Toronto Transit Commission (TTC) and Vancouver TransLink (TL). A total of 14 vehicles were used and all Cu products were installed on high-touch stanchions and handles on three buses, two subway cars, and two street cars in Toronto, as well as three buses and four SkyTrain cars in Vancouver. To ensure even use of all three Cu types, products were alternated to different stanchions and high-touch locations among the same vehicle types and their opposite stanchions were used as controls. The transit vehicles chosen were on high-traffic routes and were tested monthly for durability and every two months for antimicrobial efficacy by a third party auditing company (Westech Inc., Vancouver, British Columbia) from October 2021 to October 2022. The auditing company had trained and standardized sampling protocol in both cities. Transit vehicles traveled on their regular morning routes before returning to the depot for their audit. Microbial samples were taken one to three hours after the last passenger departed from the transit vehicle and prior to cleaning. An overview of auditing procedures can be seen in Fig. 1. In the event of a coinciding microbial and durability audit, microbial audits were performed first. Stanchions were then wiped with a disinfectant following the microbial audit to remove any residue from the microbial sampling template, the ATP SuperSnaps™(Scigiene Corp, Ontario, Canada), and/or Petrifilm agar (Remel, Kansas, USA) and allowed to dry before the durability assessment.

### Microbial outcome measurements

Numbered sampling templates were applied to Cu and control stanchions to standardize the collection of bacterial culture (colony forming units or CFU) and ATP Bioluminescence relative light units (ATPB RLU). Triplicate CFUs were collected from each high-touch surface for every Cu product and control. Limited surface area permitted only one ATPB test for each high-touch surface. All sample sites had a mirrored control (a painted carbon-steel stanchion) that was tested in parallel.

#### In-field culture sampling, colony forming unit (CFU)—3M™ Petrifilm™

Prior to every audit, 3M™ Petrifilm™ Aerobic Count Plates (PF) (3M, Ontario, Canada) were hydrated with one milliliter Letheen broth and labeled with a QR code indicating city, audit number, vehicle number, stanchion location, and culture replicate number. Laboratory-prepared PF kits were picked up by the Westech Inc. (Vancouver, British Columbia) auditing team immediately prior to audit and stored between 2 and 8 °C until used. A PF plate with hydrated media was opened and applied within the corresponding 20 cm^2^ sampling template on the test surface. The hydrate media of the PF was gently rubbed in a lateral and horizontal direction for ten seconds to ensure complete contact with the surface. The PF was then carefully removed from the template and re-covered with the protective film. Inoculated PFs were stored at room temperature after sampling and returned directly to Vancouver General or Mount Sinai Hospital laboratories for incubation at 30 °C for 48 h.

#### In-lab CFU processing

After 48 h, CFUs were enumerated using the Petrifilm Plate Reader Advanced imager and the 3M Petrifilm Plate Manager Software v2.0.1 (3M, Ontario, Canada). Ten percent of PF plates were counted manually to confirm CFU numbers. Following enumeration, a randomized table was used to select one of the PF triplicate cultures per stanchion for transfer by direct contact onto 5% sheep blood agar plates (BAPs) to permit colony identification after overnight incubation at 37 °C. Bacterial colonies with different morphologies were selected and identified using the Bruker MALDI Biotyper™ and flexControl v3.4 software (Becton Dickinson, New Jersey, USA) as well as the MBT compass reference library (version 2022) (Bruker, Ontario, Canada). Unidentified colonies from MALDI were gram stained and identified manually with traditional microbial testing. Gram negative and potentially pathogenic gram-positive colonies recovered from Cu surfaces were frozen at − 20 °C.

### In-field ATP sampling

ATP SuperSnaps^®^ were used to swab the area within each sampling template for 20 s in vertical and slightly angled horizontal directions to ensure complete coverage of the sampling area. The SuperSnap^®^ swab was then activated as per manufacturer’s instructions, shaken vigorously for ten seconds, and immediately placed into an EnSure Luminometer (Hygiena, California, USA) to obtain an ATPB reading in RLU.

### Durability outcome measurements

All durability audits described below were performed monthly and each Cu product had two durability sample sites per high-touch surface.

#### Visual damage inspection

Auditors examined all stanchions carefully for evidence of chipping, peeling, discolouration, or other damage. Photos were taken of the stanchions for visual evidence.

#### RGB reading procedure

Six Red–Green–Blue (RGB) data point readings were recorded per stanchion for detection of Cu colour changes and tarnishing. RGB readings were collected by placing the Nix Sensor (Nix Sensor Ltd, Ontario, Canada) at six evenly spaced data points along the stanchion following a helix pattern. Colour readings were recorded using the Nix ProColor app (Nix Sensor Ltd, Ontario, Canada).

#### Copper concentration

Surface Cu concentration was measured indirectly using the Waterloo Copper Concentration Kit (University of Waterloo, Waterloo, Canada). This kit applies a reagent that reacts with surface available Cu and generates a magenta colour. The intensity of the magenta color is then used to infer the surface available Cu concentration, via comparison with a calibration rod. Briefly, the measurements proceed as such: test powder and test liquid were mixed and left for five minutes. Liquid was then applied to the cotton pad of two adhesive bandages and affixed to different areas on a stanchion. Band-Aids were removed after 2 minutes, and colour readings were taken by the Nix Sensor and read using the Nix ProColor app. Stanchions were disinfected following all durability test procedures.

### Physical performance measures

The morphology and chemical composition of the as-received Cu products (i.e. unexposed) and after 12 months of installation in buses, subway cars, street cars, and SkyTrains were evaluated using scanning electron microscopy (SEM) equipped with energy-dispersive X-ray spectroscopy (EDS).

### Data analysis

CFU and ATP data were analyzed using RStudio Team^12^ software using R engine R Core Team^13^. Modeling was conducted using R Core Team^14^ and and regression model packages for model diagnostics^15–18^. Exploratory plots and data wrangling were conducted using CFU values were deemed countable as per PF instructions between 0 and 300 CFU/PF plate^19^; counts above 300 CFU/plate were replaced with the highest CFU value of 300 as per Christen and Parker statistical analysis of microbial data^20^. The primary analysis for estimating the Cu effect on CFU at 12 months was conducted using linear mixed effect model with log 10 CFU as a response, Cu/Control group as fixed effect, and random effects for City, Vehicle and Stanchion ID. For the secondary outcome, ATPB, the marginal Poisson model was used with generalized estimating equations (GEE), with ATPB as a response variable, Cu/Control group as fixed effect, and compound symmetry correlation structure of the residuals within City. Bacterial and fungal species distribution was prepared using Prism GraphPad version 9 (GraphPad, California, USA).

## Results

### Antimicrobial efficacy

**Figure 2:**
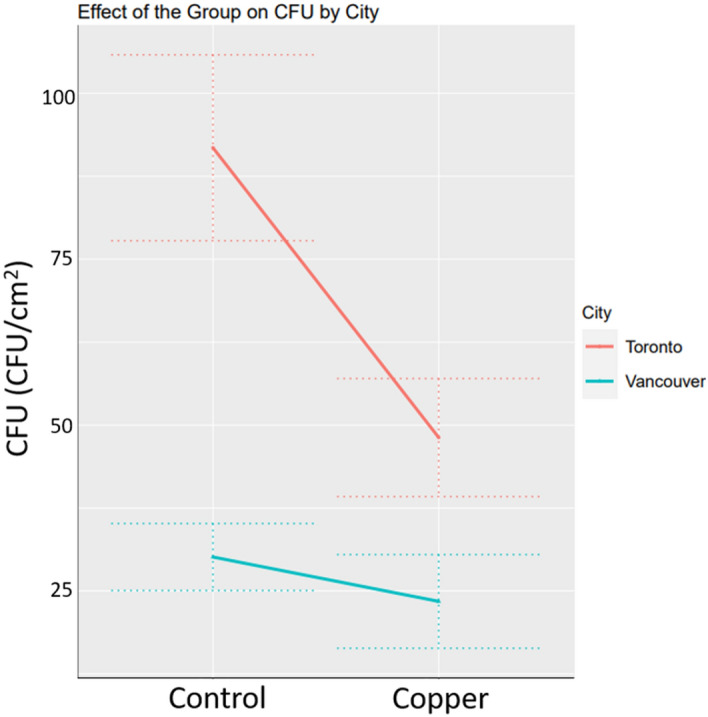
The effect of Cu compared to the control stanchions on the CFU counts after 12 months of in vehicle use.

A total of 132 pairs of microbiology sample sites and control sites were tested during this study. Microbial samples were collected every two months between the period of October 2021 to October 2022 in both Toronto and Vancouver, Canada from Cu (*n* = *2631)* and control surfaces (*n* = *2268)*. After 12 months of use in public transit, the three Cu formulations exhibited 42.7% reduction in the mean CFU (0.573 (CI 95% 0.453–0.726), p-value < 0.001) compared to control surfaces (Fig. 2). CFU counts from Toronto demonstrated a larger percent reduction of 46.8% compared to 39.1% in Vancouver, with estimated effects of (0.532(CI 95% 0.372–0.761), p-value < 0.001) and (0.609(CI 95% 0.441–0.839), p-value = 0.0028) in Toronto and Vancouver, respectively. The estimates, confidence intervals and the p-values were obtained on log 10 CFU scale; however, only the estimates and their confidence intervals are back-transformed to the original CFU scale.

### Microorganism identification

**Figure 3:**
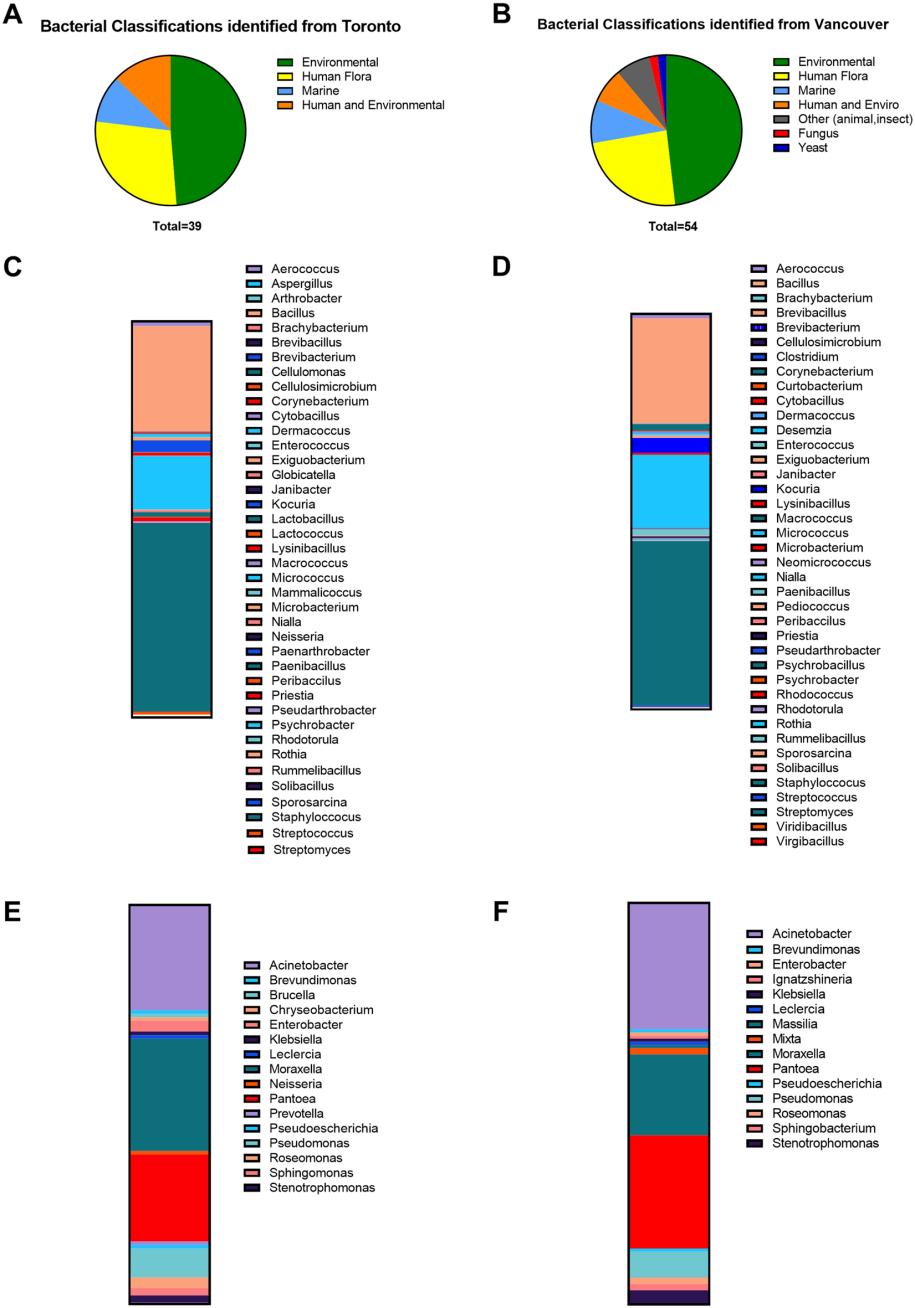
Microorganisms commonly isolated and identification by city and surface type. Gram-positive and gram-negative bacterial genera classifications from commonly isolated environments for (**A**) Toronto and (**B**) Vancouver. Genera diversity from gram-positives isolated and identified on (**C**) Control and (**D**) copper surfaces and gram-negatives on (**E**) control and (**F**) copper surfaces. Identified microorganisms were selected at random from one of the triplicate microbial samples for each stanchion surface per audit (*n* = *882; control stanchions)* and (*n* = *877; Cu stanchions)*.

Both Toronto and Vancouver (Fig. 3A,B) stanchion sites predominantly grew common environmental or human flora. Greater micro-organism diversity was noted in Vancouver and included yeast, fungi, and insect-based bacterial species not observed in Toronto. *Bacillus*, *Kocuria*, *Micrococcus*, and *Staphylococcus* spp. were the most common gram-positive organisms on all surface types (Fig. 3C,D, Supplemental Table 1–3). *Acinetobacter*, *Moraxella*, *Pantoea*, and *Pseudomonas* spp. were the most frequently isolated gram-negative genera (Fig. 3E,F).

**Figure 4:**
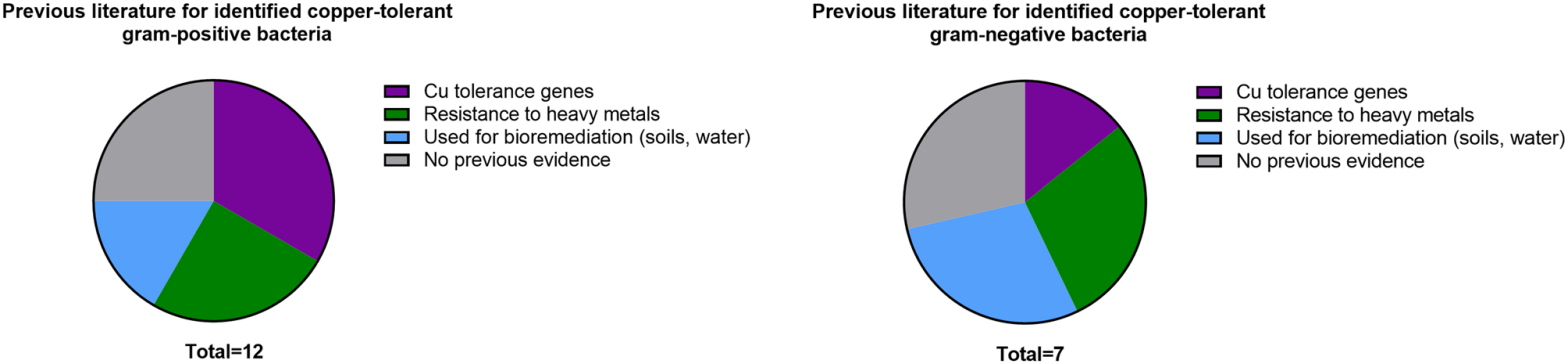
Evidence for copper-tolerance in micro-organisms isolated and identified from copper stanchions.

A literature search was conducted to determine if the hereby detected genera were previously reported as Cu-tolerant (Fig. 4, Supplemental Table 4). Upon review, twenty-one genera were noted to grow on Cu surfaces, of these, five were not previously reported as Cu-tolerant, five were noted to have heavy metal (HM) tolerance, four were described as bacteria used in bioremediation of HM contaminated soils or water sediments, and seven had previously been described as harboring a Cu-tolerance phenotype or homeostasis genes.

### ATPB readings

**Figure 5:**
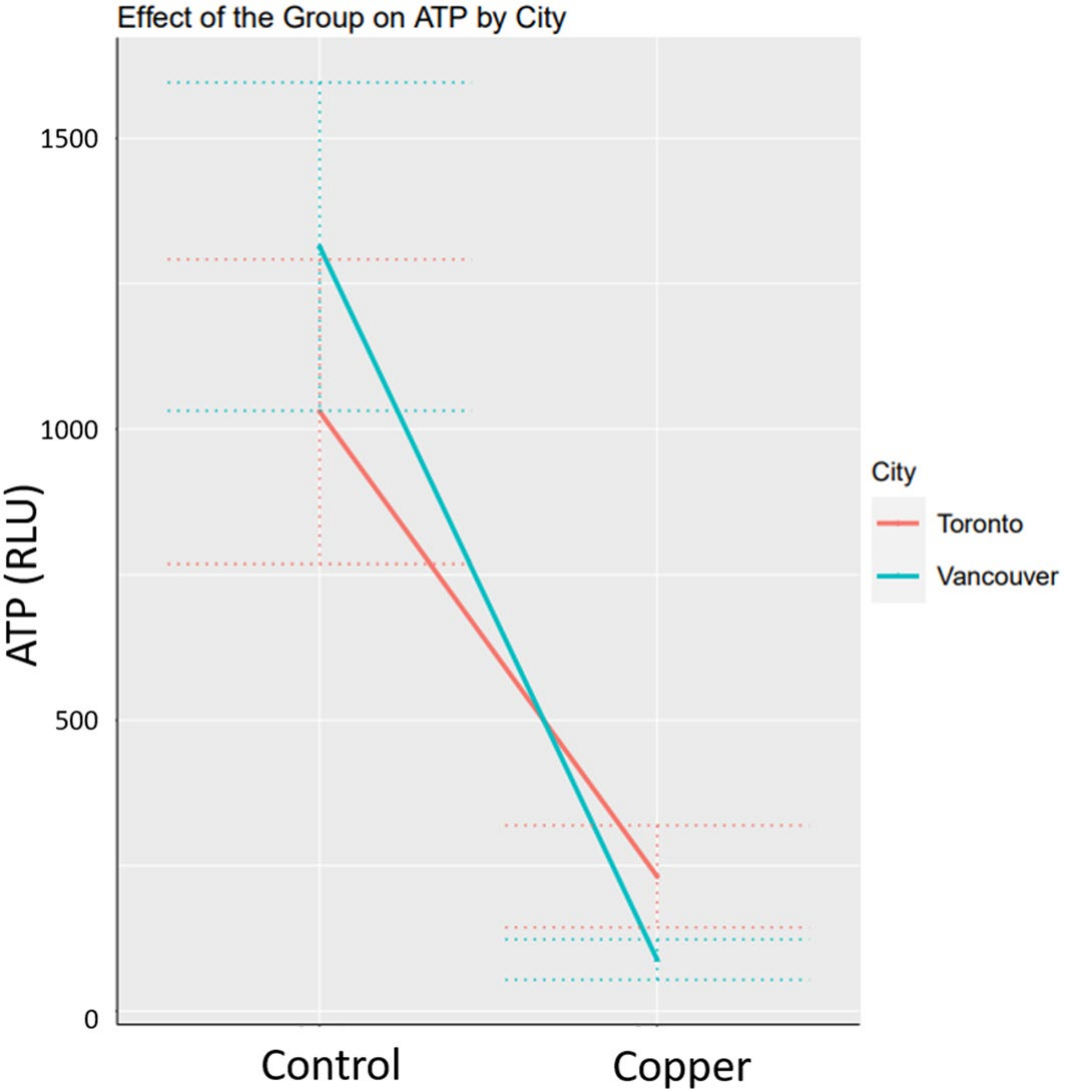
The effect of Cu compared to the control stanchions on the ATP RLU counts after 12 months of in vehicle use.

ATPB was used as an indirect measure of the organic material deposited on the stanchions. Importantly, the three Cu products overall exhibited 87.1% reduction in the mean ATPB readings (0.129 (CI 95% 0.059–0.285, p-value < 0.001) compared to controls (Fig. 5). RLU readings demonstrated a 77.5% reduction in Toronto and a 93.3% reduction in Vancouver.

Regression analysis of the effect of CFU on ATP indicated a statistically significant, but practically rather small effect size, i.e. with one unit increase in CFU, the mean ATP increases by (1(CI 95% 1–1), p-value < 0.001). At baseline (CFU = 0) the mean ATP is estimated at (553 CI 95% 414–738), p-value < 0.001).

### Physical performance of Cu

At the 12-month time point, SEM cross-sectional analysis did not detect any significant reduction in the thickness of the Cu products. Similarly, EDS elemental cross-sectional analysis (Supplemental Fig. 3) did not show any signs of dealloying. However, top-down SEM analysis (Supplemental Fig. 4) demonstrated the presence of a substantial amount of carbon-containing substance (i.e. dirt) in all three Cu products as well as the controls. This contamination was found to be distributed unevenly but was notably concentrated within surface crevices and grooves.

**Figure 6:**
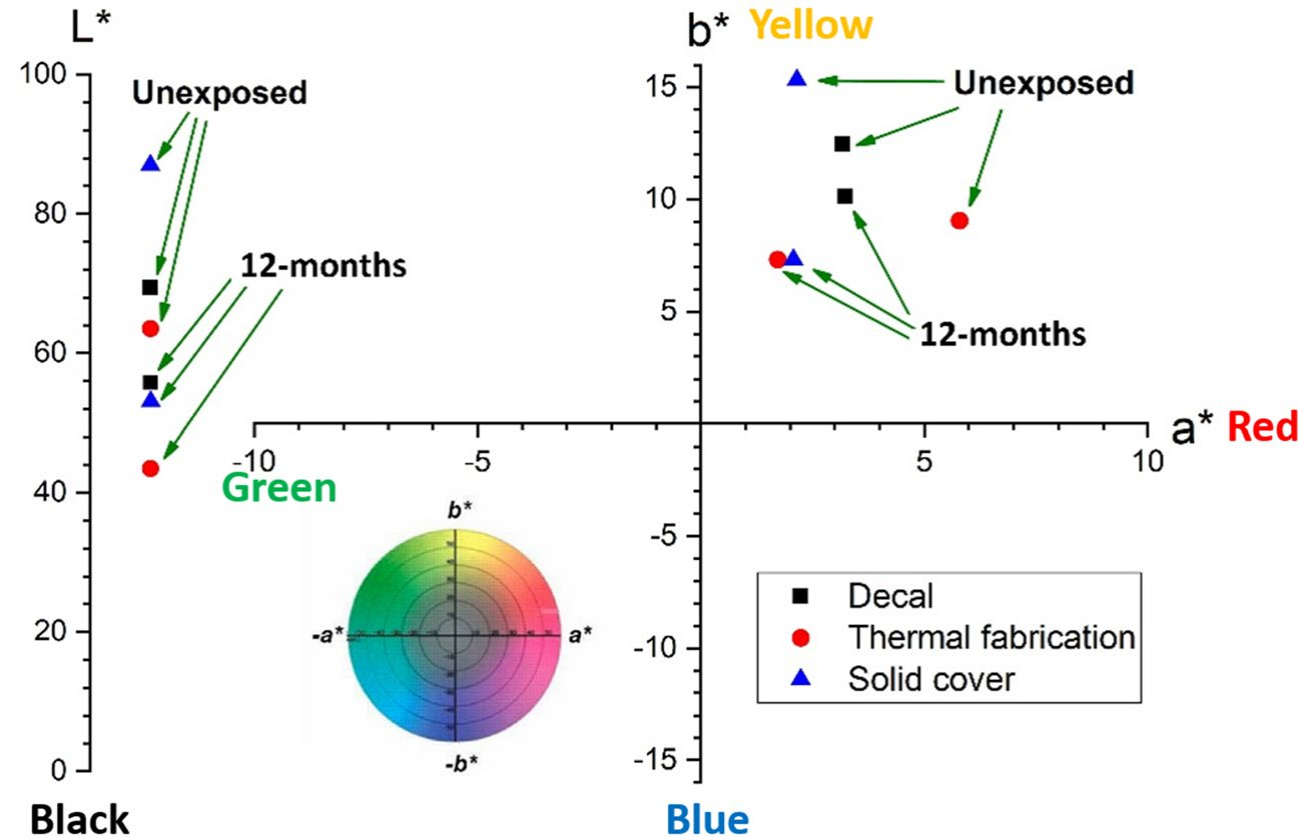
Changes in surface colour of the Cu products in the unexposed (as-received) condition and after 12-months of installation in buses, subway cars, and street cars. Surface appearance is described by the three coordinates, a* (red/magenta-green), b* (yellow-blue) and L* (lightness − black to white) in the CIELab colour reference space.

While visual inspection showed only minor changes in colouration, the colorimetry measurements revealed that all Cu products became darker (Fig. 6) after installation in all vehicles, likely due to oxidation of the copper surface and carbon-contamination.

### Surface Cu concentration

**Figure 7:**
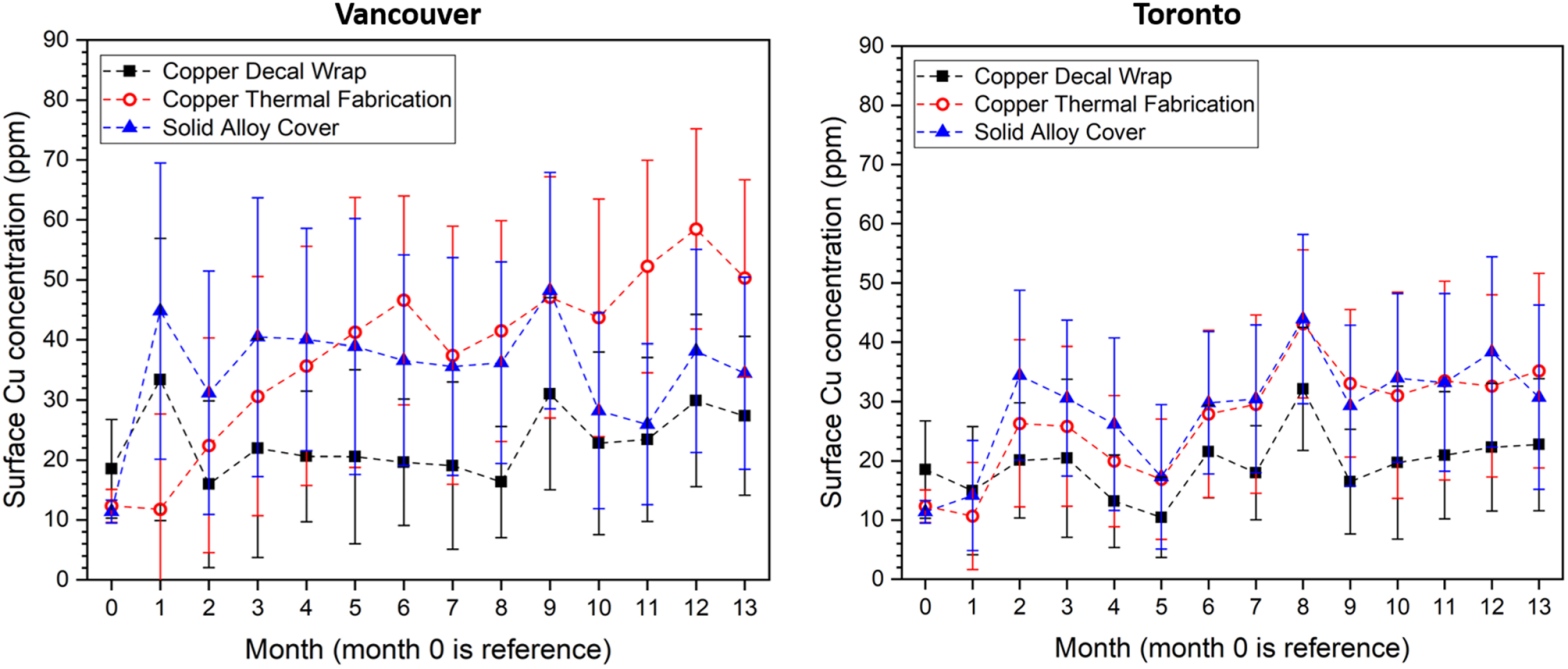
The average surface Cu concentration for three different Cu products installed in public transit systems in Vancouver and Toronto as a function of time determined from Waterloo Copper Concentration Kit.

Figure 7 illustrates the time-dependent average surface Cu concentration for the products obtained using the Waterloo Copper Concentration Kit, with the higher Cu concentration indicating an increased release of Cu ions from the product's surface. Generally, the Cu products installed in Vancouver's public transit system had higher surface Cu concentrations compared to those installed in Toronto's public transit system after one year of use.

## Discussion

Many peer-reviewed articles have demonstrated that Cu has antimicrobial properties in *in-situ* setting such as hospitals, care facilities, and university dorms^5–9^. However, the vast majority of these studies have assessed a single type of Cu product for short periods and/or in low to moderate use areas which makes it difficult to assess the benefits of using Cu as a long-term mitigation strategy particularly in the transit system or other public venues. This in-use study of three Cu products assessed both durability and antimicrobial efficacy after 12 months of hand contact and weekly cleaning/disinfection on public transportation. All vehicles used accelerated hydrogen peroxide apart from buses in British Columbia that used a quaternary ammonium product. During the course of the project, cleaning and disinfection was daily on some vehicles and changed to weekly at various times during the year-long evaluation.

This study demonstrated that Cu products on stanchions reduced the mean CFU by 42.7% compared to control surfaces across Vancouver and Toronto. It should be noted that during the pandemic both cities changed from monthly to weekly cleaning and disinfection with either accelerated hydrogen peroxide or quaternary ammonium-based products. The more frequent cleaning and the reduced ridership (world-wide use of transit dropped by up to 80% in some countries during the peak of the pandemic^21^) combined with increased glove and mask use by riders, surely reduced the CFU on all high-touch surfaces including the controls. Regardless, a statistically significant difference between Cu and control stanchions in CFU after 12 months of use was still observed. Some of the surface CFU concentrations are well above the quantification range in this study methods (i.e. above 300 CFU per 20 cm^2^ PF), and these values were censored to their highest values (300 CFU per 20 cm^2^ PF) as per^17^. The censoring could be a potential source of bias, however, due to small percentage of censored observations (≤  15% of the total data set), we expect that the estimates obtained from log-normal model used in this study is mitigated^20,22–25^.

There are large discrepancies between previous peer-reviewed studies quantifying micro-organisms on public transportation vehicles. One study completed in central London on buses, trains, stations and hotels found a median colony forming unit (CFU) count of 12 CFU/cm^2^^26^. Inversely one study completed in England and Scotland on railway stations found a max bacterial yield of 10^7^–10^8^ CFU/cm^2^ and 10^4^–10^5^ CFU/cm^2^ for Fungi^27^. A third study in Bangladesh found CFU counts on grab rails ranging from 32–263 × 10^4^ CFU/4cm^2^^28^. This study further identified enteric and pathogenic bacteria and reported from the 45 sampling sites that *E. coli* was identified on 73%, Methicillin-resistant *Staphyloccus aureus* (MRSA) on 26.6%, and *Salmonella typhi* on 37.7% of the sampling sites. Other studies in Mexico and Barcelona have identified mainly environmental and human commensal bacteria such as *Corynebacterium* spp., *Streptococcus* spp., *Staphylococcus* spp.^29^, and *Acinetobacter* spp.^30^.

There was great diversity in the isolated and identified bacterial, fungal, and yeast species from the public transit systems in both metropolitan Toronto and Vancouver. However, Vancouver was observed to have more species diversity compared to Toronto that included environmental bacteria, yeast, and fungal species. This could be attributed to the different climates between the two cities or possibly to the cleaning methods used between the sites. As expected the bacteria isolated were primarily gram-positive and likely reflected their relative impermeability to Cu conferred by their thicker peptidoglycan cell walls^31^ The vast majority (99.6%) of bacteria were commensal environmental or human flora but did include some potential pathogens such as *Staphylococcus aureus—*an opportunistic pathogen with high transmission rates and the potential for antibiotic resistance^32^. Of the gram-negative species of bacteria found on Cu, most were soil and water commensals that had previously been described in soils contaminated with heavy metals^33–35^. Cu resistance was only previously reported in 0.7% of isolated and identified bacteria in the transit study. The possibility of Cu resistance over time with subsequent transfer of Cu resistance genes cannot be excluded, however, literature reports of Cu resistance from items containing this metal (e.g. coins, fixtures, jewelry) are rare^36^. Moreover, allergic reactions to Cu are very uncommon and generally reflect reactions to other metals such as zinc or nickel contained in Cu alloys^37^.

The secondary microbial outcome of this study, ATPB analysis, found significant reductions in organic material on Cu compared to control stanchions. CFU counts reflect only common bacteria that can be cultivated in a clinical microbiology laboratory while ATPB assesses organic material from all matter—living or dead. This secondary measure provides valuable supporting information to CFU measurements for total non-cultivable organisms. In concordance with the observed difference in species diversity between Vancouver and Toronto, ATPB RLU values were higher in Vancouver. Danko et al. have recently demonstrated that the climate and geographic characteristics were high predictors of taxonomic diversity in the public transportation microbiome^38^.

All Cu products maintained similar surface Cu concentration throughout the duration of study while demonstrating only minor discolouration during the 12-month trial. However, certain Cu applications were more vulnerable to vandalism (removal, peeling, ripping), dirt (i.e. carbon accumulation), or tarnishing. Regardless, all surfaces showed constant or higher surface Cu concentrations over time. The surface Cu concentrations were higher in Vancouver, consistent with a higher humidity and warmer environment – metal dissolution processes are faster at more elevated temperatures and in the presence of moisture^39^. The actual temperatures and humidity levels of the stanchions’ surfaces while in service were logistically impossible to monitor in this trial. Surface analysis (for example, X-Ray Photoelectron Spectroscopy) of the products formed on the stanchions was not attempted as surface conditions would change from the point of collection to analysis and would render any results of the near surface (at the nanometer scale) unrepresentative. However, deeper penetration X-Rays from the EDS system (micrometer scale) did yield chlorides indicating at least partial formation of Cu-chloride species that are detected in atmospheric corrosion of Cu. Most importantly, although dirt accumulation was noted on all the stanchion surfaces, Cu release at the surface and thus its anti-microbial effect(s) was not hindered.

A limitation of this study was the inability to collect viral samples. While viral sampling possibly could have been correlated to the SARS-CoV2 waves and level of restrictions, it was both logistically and technically difficult to perform. To address this, the authors performed in vitro viral and bacterial studies to assess the antimicrobial efficacy after 200-rounds of simulated cleaning (accepted for publication) and found that Cu maintained not only bactericidal but virucidal activity. During the study period, vandalism of copper stanchion and theft attempt were observed, the cost and perceived value of copper might have been incentive.

As previously mentioned, CFU counts were much lower on control stanchions than previously published likely reflecting the reduced ridership observed due to the pandemic (Supplemental Fig. 1). It was not feasible to quantify hand contact with each stanchion type to confirm this theory. Finally as responses to the pandemic changed, the cleaning schedules in the transit were altered although the basic protocols remained the same.

## Conclusion

This study evaluated the durability and antimicrobial ability of three commercially available Cu products in public transit vehicles in two Canadian cities over a 12-month period. Overall, each Cu alloy demonstrated good physical durability and continuous surface Cu concentrations that aligned with significant antimicrobial efficacy. The use of ATPB as a secondary outcome for antimicrobial efficacy further illustrated the self-sanitizing capacity of Cu products. CFU and ATPB assessments support the installation of Cu products in high-traffic environments especially when daily cleaning and disinfection is impossible. This mitigation strategy continuously reduced the bioburden on high-touch surfaces, and could result in a “biologically” safer commute for transit users.

### Supplementary Information

Supplementary Information.

## Data Availability

All data generated or analysed during this study are included in this published article [and its supplementary information files].

## Abbreviations

CFU: Colony forming units
Cu: Copper
EPA: Environmental protection agency
HM: Heavy metal
PF: Petrifilm
PMRA: Pest management regulatory agency
