# Supplementary material for: One-year trial evaluating the durability and antimicrobial efficacy of copper in public transportation systems
Source: Sci Rep. 2024 Mar 21;14:6765. doi: 10.1038/s41598-024-56225-9 (PMC10958017; doi:10.1038/s41598-024-56225-9)
Supplement: Supplementary file 1 — Supplementary Information. [file 41598_2024_56225_MOESM1_ESM.docx]

Supplemental Figures and Tables


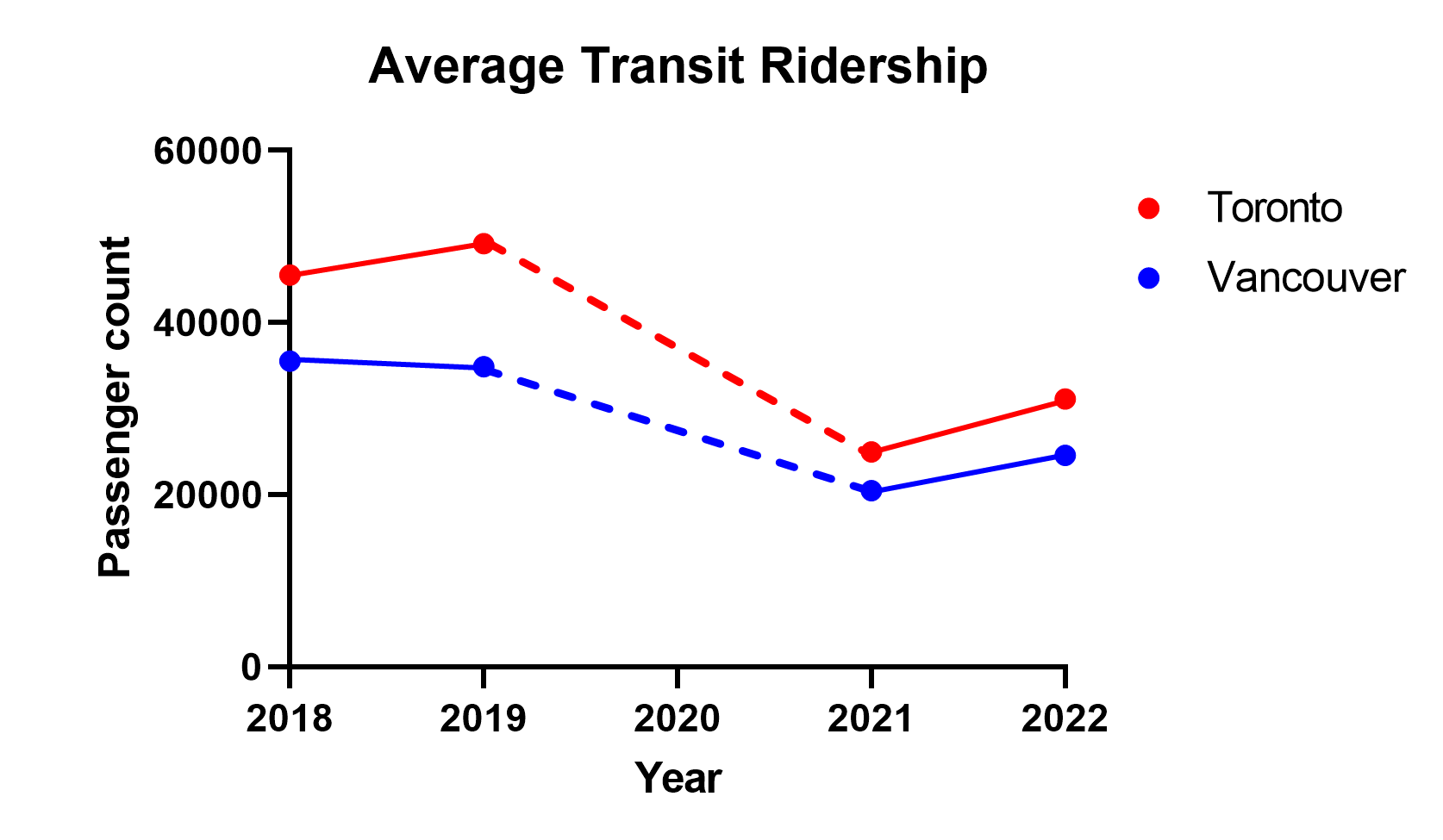


**Supplemental figure 1**: Average annual passenger count on transit vehicles for Toronto and Vancouver between the years 2018 and 2022. The dashed line indicates the estimated recovery of ridership during the SARS-CoV2 pandemic.

| **Supplemental Table 1: Common isolated bacteria recovered from public transit vehicles in Vancouver and Toronto** | |
| --- | --- |
| ***Gram Positive*** | |
| **genus** | **species** |
| *Aerococcus* | *viridans* |
| *Bacillus* | *altitudinis, cereus, circulans, clausii, flexus, horneckiae, lichenformis, megaterium, simplex, sutilis* |
| *Cellulosimicrobium* | *cellulans* |
| *Corynebacterium* | *aurimucosum, mucifaciens/ureiclerivorans* |
| *Enterobacter* | *cloacae compplex* |
| *Enterococcus* | *casseliflavus, faecalis* |
| *Exiguobacterium* | *aurantiacum* |
| *Kocuria* | *palustris, rhizophlia* |
| *Lysinibacillus* | *fusiformis* |
| *Micrococcus* | *luteus* |
| *Paenibacillus* | *lautis, pabuli, provencensis* |
| *Solibacillus* | *silvestris* |
| *Staphyloccocus* | *aureus, auricularis, capitis, cohnii, epidermidis, haemolyticus, hominis, lugdunensis, pasteuri, pattenkoferi, saprophyticus, schleiferi, sciuri, simulans, warneri, xylosus* |
|  |  |
| *Streptococcus* | *mitis/oralis, parasanguinis* |
| ***Gram Negative*** | |
| **genus** | **species** |
| *Acinetobacter* | *Iwoffii, pittii, radioresistens, ursingii* |
| *Brevundimonas* | *dimunta* |
| *Leclercia* | *adecarboxylata* |
| *Moraxella* | *osloensis* |
| *Pantoea* | *agglomerans* |
| *Pseudoescherichia* | *vulneris* |
| *Stenotrophomonas* | *maltophilia, rhizophila* |

**OPTIONAL SUPPLEMENTAL TABLES**

| **Supplemental Table 2: Bacteria isolated and identified from Toronto buses, streetcars, and subway** | |
| --- | --- |
| ***Gram Positive*** | |
| **genus** | **species** |
| *Arthrobacter* | *globiformis* |
| *Bacillus* | *firmus, idriensis* |
| *Clostridium* | *tertium* |
| *Corynebacterium* | *durum* |
| *Curtobacterium* | *pusillum* |
| *Cytobacillus* | *firmus* |
| *Exiguobacterium* | *acetylicum, hominis* |
| *Kocuria* | *carniphila* |
| *Mammalicoccus* | *sciuri* |
| *Microbacterium* | *arborescens, flavescens/laevaniformans, paraoxydans, testaceum* |
| *Nialla* | *circulans* |
| *Peribaccilus* | *simplex* |
| *Prestia* | *flexa, megaterium* |
| *Psychrobacter* | *faecalis* |
| *Rhodococcus* | *hoagii* |
| *Staphyloccoccus* | *arlettae* |
| *Stomatococcus* | *sp.* |
| *Virgibacillus* | *proomii* |
| ***Gram Negative*** | |
| *Acinetobacter* | *baumannii, johnsonii* |
| *Brucella* | *anthropi* |
| *Klebsiella* | *oxytoca* |
| *Prevotella* | *oris* |
| *pseudomonas* | *aeruginosa, fluorescens, luteola, oryzihabitans, putida, stutzeri* |

| **Supplemental Table 3: Bacteria isolated and identified from Vancouver skytrain and busses** | |
| --- | --- |
| ***Gram Positive*** | |
| **genus** | **species** |
| *Aerococcus* | *sanguinicola* |
| *Aspergillus* | *fumigatus* |
| *Bacillus* | *amyloliquifaciens, badius, fordii, galactosidilyticus, halodurans, halosaccharovorans, horikoshii, humi, infantis, lentus, marisflavi, myocoides, oceanisediminis, oleronius, pumilus, shackletonii, sonorensis, thermoamylovarans, vietnamensis* |
| *Brachybacterium* | *conglomeratum, muris, nesterenkovii, rhamnosum* |
| *Brevibacillus* | *centrosporus* |
| *Cellulomonas* | *pakistanensis* |
| *Corynebacterium* | *afermentans, amycolatum, callunae, casei, coyleae, imitans, provenscense* |
| *Curtobacterium* | *Flaccumfaciens* |
| *Dermacoccus* | *nishinomiyaenesis* |
| *Desemzia* | *incerta* |
| *Enterococcus* | *facium* |
| *Globicatella* | *sanguinis* |
| *Janibacter* | *hoylei* |
| *Kocuria* | *kristinae, marina, salsicia* |
| *Lactobacillus* | *coleohominis, sakei* |
| *Lactococcus* | *garvieae* |
| *Lysinibacillus* | *halotolerans, massiliensis, sphaericus, xylanilyticus* |
| *Macrococcus* | *canis* |
| *Micrococcus* | *endophyticus, flavus, terreus* |
| *Microbacterium* | *esteraromaticum, oleivorans, oxydans,* |
| *Neomicrococcus* | *lactis* |
| *Paenarthrobacter* | *aurescens, nicotinovorans* |
| *Paenibacillus* | *amylolyticus, cineris, etheri, glucanolyticus, illionisensis, lactis, pasadenesis, pheonicis, rhizosphaerae, tylopili, urinalis, woosongensis* |
| *Pediococcus* | *pentosaceus* |
| *Pseudoarthrobacter* | *oxydans, polychromogenes* |
| *Psychrobacillus* | *psychrodurans* |
| *Rhodotorula* | *mucilaginosa* |
| *Rothia* | *terrae* |
| *Rummelibacillus* | *pycnus, stabekisii* |
| *Sporosarcina* | *luteola, aquimarina* |
| *Staphyloccocus* | *caprae, condimenti, equorum, nepalensis, petrasii, pseuditermedius, succinus* |
| *Streptococcus* | *gordonii, peroris, pneumoniae, sanguinis, vestibularis* |
| *Streptomyces* | *nogalater, violaceoruber* |
| *Viridibacillus* | *neidei* |
| ***Gram Negative*** | |
| *Acinetobacter* | *calcoaceticus, nosocomialis, pseudoIwoffii* |
| *Chryseobacterium* | *hominis, ureilyticum* |
| *Ignatzshineria* | *indica* |
| *Massilia* | *varians* |
| *Mixta* | *calida* |
| *Neisseria* | *subflava* |
| *Pantoea* | *eucrina, septica, vagans* |
| *Pseudomonas* | *fulva, koreensis* |
| *Roseomonas* | *mucosa* |
| *Sphingobacterium* | *multivorum* |
| *Sphingomonas* | *parapaucimobilis, paucimobilis* |

**Supplemental Table 4: Copper-tolerance micro-organisms isolated and identified from copper stanchions**

| **Genus** | **Previously referenced Cu-tolerance** | **Reference** |
| --- | --- | --- |
| *Cu-resistant gram positive genera* | | |
| *Clostridium* sp. | Cu tolerance genes | [1] |
| *Corynebacterium* sp. | Cu tolerance genes | [2] |
| *Curtobacterium* sp. | none | - |
| *Desemzia* sp. | none | - |
| *Ignatzshineria* sp. | none | - |
| *Pediococcus* sp. | Cu homeostasis genes | [3, 4] |
| *Pseudarthrobacter* sp. | Cu tolerance genes | [5] |
| *Psychrobacillus* sp. | resistance to HM | [6, 7] |
| *Psychrobacter* sp. | resistome profile | [8] |
| *Rhodococcus* sp. | resistance to HM | [9] |
| *Rothia* sp. | resistance to HM | [10] |
| *Virgibacillus* sp. | remediation of HM contaminated marine sediment | [11] |
| *Viridibacillus* sp. | bioremediation of HM contaminated soils | [12] |
| *Cu-resistant gram negative genera* | | |
| *Brevundimonas* sp. | Phytoremediation of HM contaminated soils | [13, 14] |
| *Leclercia* sp. | Cu tolerance genes | [15, 16] |
| *Mixta* sp. | none | - |
| *Pseudoescherichia* sp. | none | - |
| *Pseudomonas* sp. | HM contaminated soils and water, hospitals, produce | [16-19] |
| *Sphingobacterium* sp. | HM contaminated soils | [20] |
| *Stenotrophomonas* sp. | Phytoremediation of HM contaminated soils, Cu resistance genes | [14, 19, 20] |


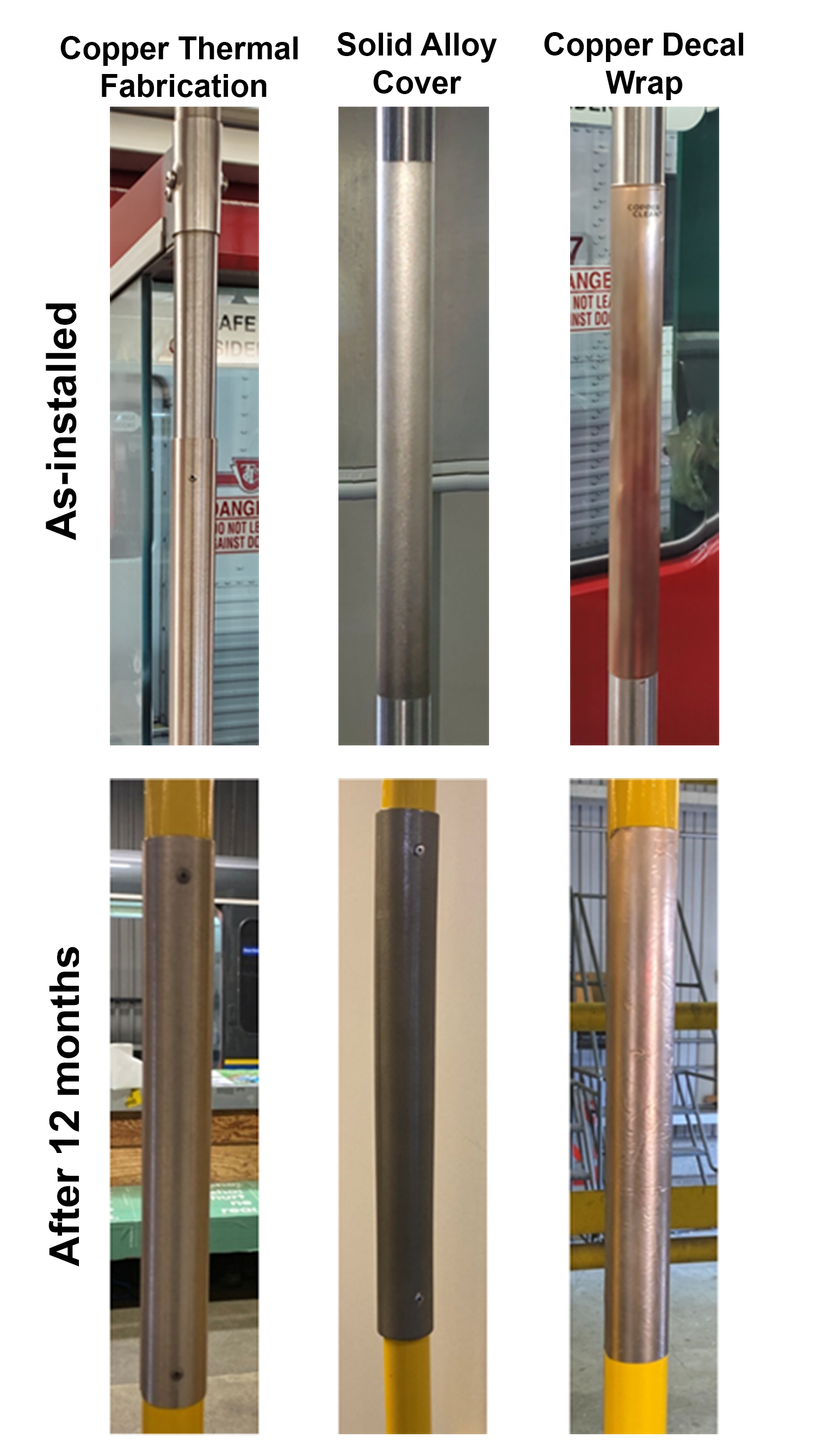


**Supplemental Figure 2.** Images of the copper stanchions just after installation and after 12 months of installation, where the stanchion is getting darker, possibly due to oxidation or carbon-contamination of the stanchion resulting from daily usage.


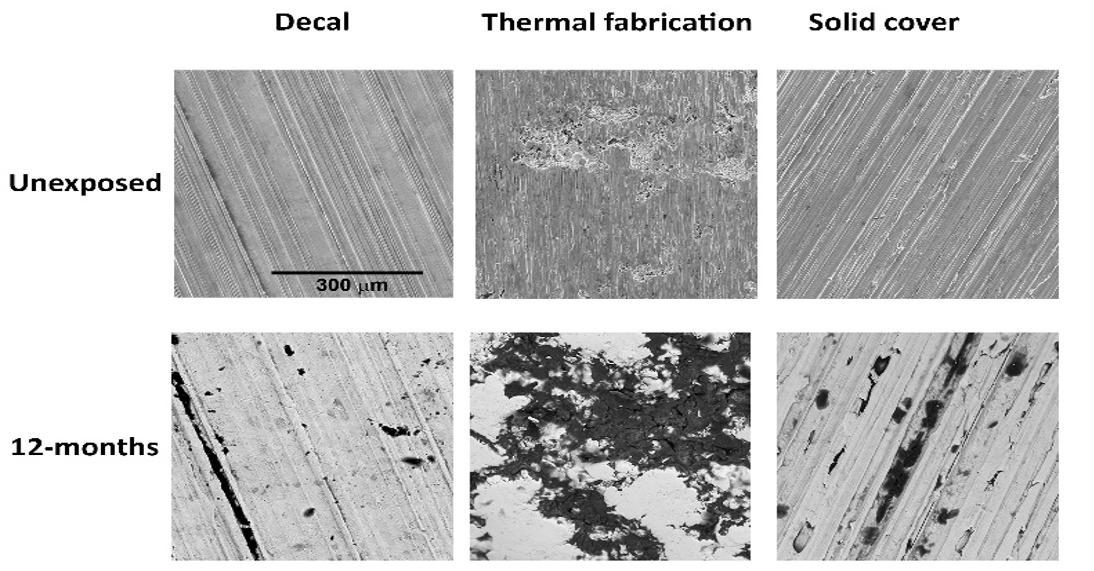


**Supplemental Figure 3.** Top-down SEM images of the copper surfaces. Top-down SEM images of the copper surfaces used for 12 months reveal the presence of a carbon-containing substance, which can be seen as dark regions on the surface. The carbon contamination was observed to be more concentrated in surface crevices and grooves, potentially reducing the effective copper surface area and affecting the copper release behavior. Note that the scratches visible on the Decal and Solid cover are a result of the production process and are oriented perpendicular to the long axis of the stanchion.


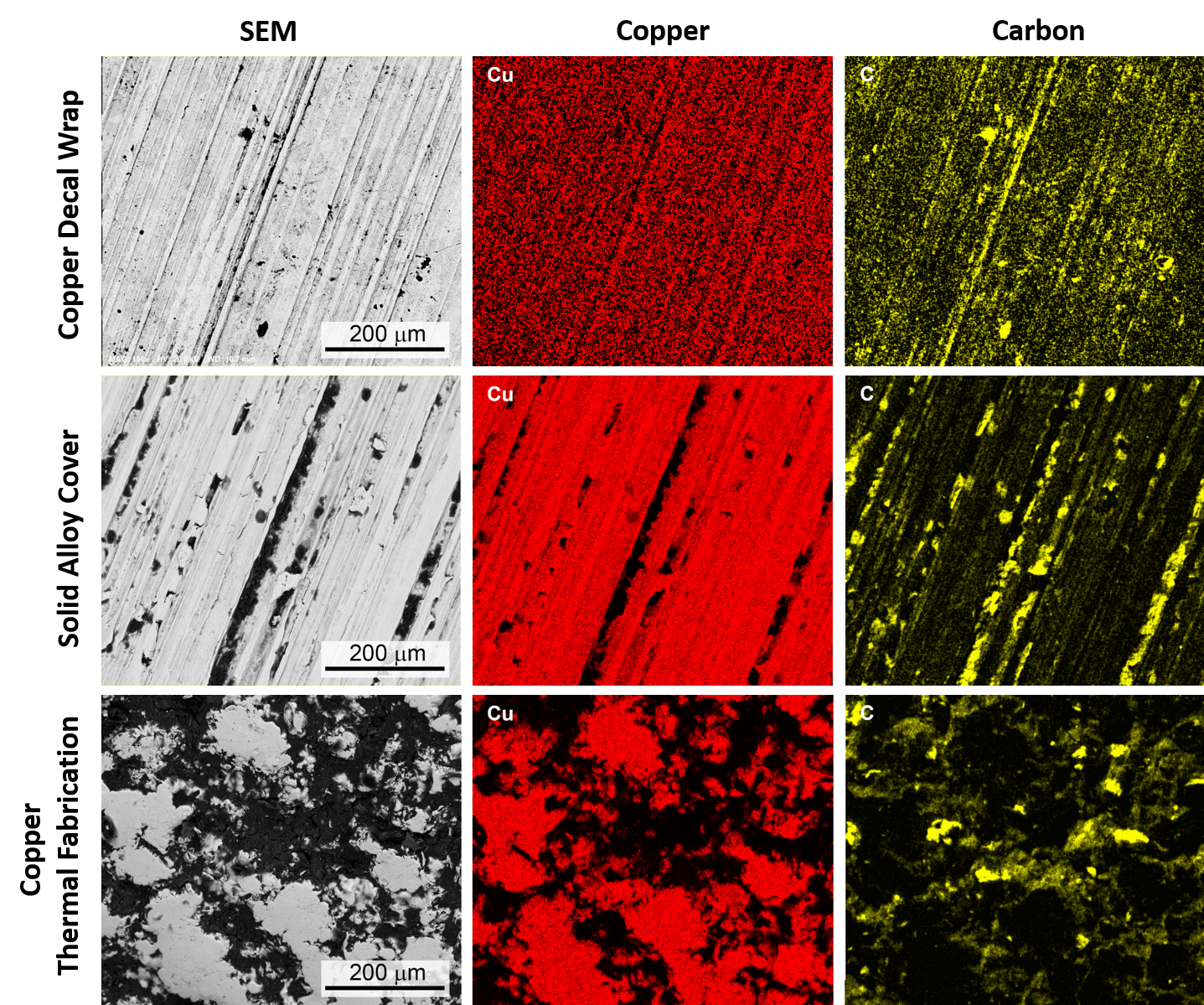


**Supplemental figure 4: SEM/EDS analysis of Cu coatings after 12 months of use**

**Supplemental References:**

1. Zhou Q, Zhou T, Feng F, Huang S, Sun Y: **The response of copper resistance genes, antibiotic resistance genes, and intl1/2 to copper addition during anaerobic digestion in laboratory**. *Ecotoxicology and Environmental Safety* 2021, **210**:111822.

2. Ladomersky E, Petris MJ: **Copper tolerance and virulence in bacteria**. *Metallomics* 2015, **7**(6):957-964.

3. Feng P, Yang J, Zhao S, Ling Z, Han R, Wu Y, Salama E-S, Kakade A, Khan A, Jin W *et al*: **Human supplementation with Pediococcus acidilactici GR-1 decreases heavy metals levels through modifying the gut microbiota and metabolome**. *npj Biofilms and Microbiomes* 2022, **8**(1):63.

4. Wanna W, Surachat K, Kaitimonchai P, Phongdara A: **Evaluation of probiotic characteristics and whole genome analysis of Pediococcus pentosaceus MR001 for use as probiotic bacteria in shrimp aquaculture**. *Scientific Reports* 2021, **11**(1):18334.

5. Mesa-Marín J, Pérez-Romero JA, Redondo-Gómez S, Pajuelo E, Rodríguez-Llorente ID, Mateos-Naranjo E: **Impact of Plant Growth Promoting Bacteria on Salicornia ramosissima Ecophysiology and Heavy Metal Phytoremediation Capacity in Estuarine Soils**. *Frontiers in Microbiology* 2020, **11**.

6. Cao J, Nagl S, Kothe E, Köhler JM: **Oxygen sensor nanoparticles for monitoring bacterial growth and characterization of dose–response functions in microfluidic screenings**. *Microchimica Acta* 2015, **182**(1):385-394.

7. Kato C, Honma A, Sato S, Okura T, Fukuda R, Nogi Y: **Poly 3-hydroxybutyrate-co-3-hydroxyhexanoate films can be degraded by the deep-sea microbes at high pressure and low temperature conditions**. *High Pressure Research* 2019, **39**(2):248-257.

8. Centurion VB, Delforno TP, Lacerda-Júnior GV, Duarte AWF, Silva LJ, Bellini GB, Rosa LH, Oliveira VM: **Unveiling resistome profiles in the sediments of an Antarctic volcanic island**. *Environmental Pollution* 2019, **255**:113240.

9. Adhami E, Aghaei SS, Zolfaghari M-R: **Evaluation of Heavy Metals Resistance in Biofilm Cells of Native Rhodococcus spp. Isolated from Soil**. *Archives of Hygiene Sciences* 2017, **6**(3).

10. Evangelista J, Conrad D: **A Case Report Of The Metagenomic Analysis Of A Patient Undergoing A Cystic Fibrosis Exacerbation**. In: *D25 PULMONARY INFECTIONS AND TUBERCULOSIS: CASE REPORTS OF NOTE.* edn.: American Thoracic Society; 2012: A5472-A5472.

11. Besaury L, Marty F, Buquet S, Mesnage V, Muyzer G, Quillet L: **Culture-dependent and independent studies of microbial diversity in highly copper-contaminated Chilean marine sediments**. *Microbial ecology* 2013, **65**:311-324.

12. Kang C-H, Kwon Y-J, So J-S: **Bioremediation of heavy metals by using bacterial mixtures**. *Ecological Engineering* 2016, **89**:64-69.

13. Rathi M, K N Y: **Brevundimonas diminuta MYS6 associated Helianthus annuus L. for enhanced copper phytoremediation**. *Chemosphere* 2021, **263**:128195.

14. Rathi M, Nandabalan YK: **Copper-tolerant rhizosphere bacteria—characterization and assessment of plant growth promoting factors**. *Environmental Science and Pollution Research* 2017, **24**(10):9723-9733.

15. Sano E, Fontana H, Esposito F, Cardoso B, Fuga B, Costa GCV, Bosqueiro TCM, Sinhorini JA, Orico LD, de Masi E *et al*: **Genomic analysis of fluoroquinolone-resistant Leclercia adecarboxylata carrying the ISKpn19-orf-qnrS1-ΔIS3-blaLAP-2 module in a synanthropic pigeon, Brazil**. *Journal of Global Antimicrobial Resistance* 2023, **33**:256-259.

16. Cidre I, Pulido RP, Burgos MJG, Gálvez A, Lucas R: **Copper and Zinc Tolerance in Bacteria Isolated from Fresh Produce**. *Journal of Food Protection* 2017, **80**(6):969-975.

17. Virieux-Petit M, Hammer-Dedet F, Aujoulat F, Jumas-Bilak E, Romano-Bertrand S: **From Copper Tolerance to Resistance in Pseudomonas aeruginosa towards Patho-Adaptation and Hospital Success**. *Genes* 2022, **13**(2):301.

18. Teitzel GM, Geddie A, Long SKD, Kirisits MJ, Whiteley M, Parsek MR: **Survival and Growth in the Presence of Elevated Copper: Transcriptional Profiling of Copper-Stressed <i>Pseudomonas aeruginosa</i>**. *Journal of Bacteriology* 2006, **188**(20):7242-7256.

19. Avanzi IR, Gracioso LH, Baltazar MdPG, Karolski B, Perpetuo EA, do Nascimento CAO: **Rapid bacteria identification from environmental mining samples using MALDI-TOF MS analysis**. *Environmental Science and Pollution Research* 2017, **24**(4):3717-3726.

20. Glibota N, Grande Burgos MJ, Gálvez A, Ortega E: **Copper tolerance and antibiotic resistance in soil bacteria from olive tree agricultural fields routinely treated with copper compounds**. *Journal of the Science of Food and Agriculture* 2019, **99**(10):4677-4685.
